# The Value of Pre-Ablative I-131 Scan for Clinical Management in Patients With Differentiated Thyroid Carcinoma

**DOI:** 10.3389/fendo.2021.655676

**Published:** 2021-05-28

**Authors:** Trynke van der Boom, Wouter T. Zandee, Claire C. J. Dekkers, Anouk N. A. van der Horst-Schrivers, Liesbeth Jansen, Schelto Kruijff, Adrienne H. Brouwers, Thera P. Links

**Affiliations:** ^1^Department of Internal Medicine, Division of Endocrinology, University Medical Center Groningen, University of Groningen, Groningen, Netherlands; ^2^Department of Surgery, University Medical Center Groningen, University of Groningen, Groningen, Netherlands; ^3^Department of Nuclear Medicine and Molecular Imaging, University Medical Center Groningen, University of Groningen, Groningen, Netherlands; ^4^Department of Emergency Medicine, Maastricht University Medical Center and Maastricht University, Maastricht, Netherlands

**Keywords:** postoperative I-131 diagnostic scan, differentiated thyroid carcinoma, uptake, clinical management, thyroid cancer

## Abstract

**Background:**

A diagnostic I-131 (Dx) scan is used to detect a thyroid remnant or metastases before treatment of differentiated thyroid cancer (DTC) with I-131. The aim of this study is to specify in which patients with DTC a Dx scan could have an additional value, by studying the effect of the Dx scan on clinical management.

**Methods:**

Patients with DTC, treated with I-131 after thyroidectomy were included in this retrospective cohort study. Twenty-four hours after administration of 37 MBq I-131 a whole body Dx scan and an uptake measurement at the original thyroid bed were performed. Outcomes of the Dx scan and the subsequent changes in clinical management, defined as additional surgery or adjustment of I-131 activity, were reported. Risk factors for a change in clinical management were identified with a binary logistic regression.

**Results:**

In 11 (4.2%) patients clinical management was changed, including additional surgery (n=5), lowering I-131 activity (n=5) or both (n=1). Risk factors for a change in clinical management were previous neck surgery (OR 5.9, 95% CI: 1.4-24.5), surgery in a non-tertiary center (OR 13.4, 95% CI: 2.8 – 63.8), TSH <53.4 mU/L (OR 19.64, 95% CI: 4.94-78.13), thyroglobulin ≥50.0 ng/L (OR 7.4, 95% CI: 1.6-34.9) and free T4 ≥4.75 pmol/L (OR 156.8, 95% CI: 128.4-864.2)

**Conclusion:**

The Dx scan can potentially change clinical management before treatment with I-131, but the yield is low. A Dx-scan should only be considered for patients with a high pre-scan risk of a change in management, based on patient history and prior center-based surgical outcomes.

## Introduction

For many years, the standard treatment for differentiated thyroid carcinoma (DTC) consisted of thyroidectomy, radio-active iodine (RAI) remnant ablation and/or (adjuvant) therapy, and TSH suppression therapy (1, 2). Nowadays, there is a trend towards de-escalation of treatment for patients with DTC, illustrated by new protocols with decreased I-131 activity for low- and intermediate risk patients (1, 3).

Since the implementation of recent guidelines, the role of the I-131 diagnostic scan (Dx scan) is being questioned. The Dx scan is used to detect a thyroid remnants or unknown lymph node metastases, potentially resulting either in pre-RAI additional surgery or a change in the administered I-131 activity (4). Furthermore, a local increased uptake at the original thyroid bed reflects the iodine uptake capacity of the thyroid remnant, which may result in an inflammatory reaction after I-131 therapy. The incidence and severity of this radiation-induced inflammation at the original thyroid bed is directly related to the uptake. This inflammation can be avoided by lowering the administered I-131 activity or by preventive prescription of anti-inflammatory drugs such as NSAIDs or corticosteroids (5). Previous literature reported additional findings on the Dx scan like unknown metastases or a remnant in 25-53% of DTC patients (6–8). However, whether these findings of the Dx scan actually changes clinical management remains unclear. Ideally, a Dx-scan should not be necessary when the quality of pre-operative staging and surgery itself is high, minimalizing the risk of a thyroid remnant or local metastases (9).

By studying the Dx scan in a specialized tertiary hospital including referrals from low-volume centers, we aim to examine the changes in clinical management based on the results of the Dx scan and to specify in which patients, if any, a Dx scan has additional value.

## Material and Methods

### Study Population

In this retrospective study, all DTC patients treated with I-131 from January 2005 until July 2015 at the University Medical Centre Groningen (UMCG) were included. Patients were excluded when a Dx scan was not performed or when recombinant human TSH (rhTSH) was used before RAI therapy. According to Dutch law, i.e. Medical Research Involving Human Subjects (WMO), no ethical review was necessary for retrospective data collection.

### Diagnostic and Treatment Protocol

In patients with a palpable thyroid nodule, ultrasonography guided fine needle aspiration cytology was performed, either at the UMCG or at the referring non-tertiary hospital. At the same session, ultrasound characteristics of the thyroid nodule were described and screening for metastatic lymph nodes in the neck region took place. All patients included in this study underwent a thyroidectomy with lymph node dissection if indicated, either at the UMCG or at one of the 19 referring hospitals. The seventh edition of the AJCC cancer staging system was used (10). Patients were classified as low risk (age 20-45 years, minimally invasive follicular carcinoma or classic papillary carcinoma, T1-2N0M0, without thyroglobulin-antibodies) or high risk (all other patients), according to the Dutch risk stratification at that time (2). In addition, patients were retrospectively classified according to a simplified ATA risk stratification as low risk (Tx-T2, Nx-N0 and Mx-M0), intermediate risk (any T3 or N1 tumor) or high risk (any T4 or M1 tumor) (1). The hospitals where the surgery was performed were classified as tertiary- (high-volume specialized hospitals) vs. non-tertiary hospitals (low-volume non-specialized hospitals). In addition, information about the surgical procedure was obtained to assess whether the thyroidectomy was performed in one or two sessions (‘one-step’ versus ‘two-step’ procedure).

After thyroidectomy, patients did not start thyroid hormone substitution and they followed a low-iodine diet starting one week prior to the RAI therapy that was generally applied 4-5 weeks after thyroidectomy. Repeat ultrasonography was not performed before the first RAI therapy. High risk patients received 5550 MBq I-131 and low risk patients received 3700 MBq I-131, according to the, at that time applicable, Dutch risk stratification criteria (11). RAI therapy was administered at discretion of the treating physician, but generally when the Dx scan at 24h showed no previously unknown metastases and an uptake < 10%. In cases with undetectable Tg and no uptake on the Dx scan, RAI therapy was still administered. TSH suppression therapy was started after RAI therapy to minimize potential tumor growth (12). Post-therapy planar whole-body scans were obtained at 7 days (from 2010 onwards, and combined with a SPECT/CT of the neck) or 10 days (before 2010) after administration of RAI therapy. Six months after RAI therapy, a 74 MBq I-131 whole body scan after 72h was performed, together with measurement of a TSH-stimulated serum thyroglobulin for re-stratification and to evaluate the I-131 therapy success.

Free T4 and TSH were measured at the time of the Dx scan, using immunochemiluminometric E-module assay (Roche Diagnostics, Indianapolis, IN, USA) with normal values of respectively 11.0 - 19.5 pmol/L and 0.5–4.0 mU/l. For the measurement of thyroglobulin (Tg), the Tg immunoradiometric assay (Tg-IRMA) by Thermo Fisher Scientific (Henningsdorf, Germany) was used, with a limit of detection of 0.1 ng/mL and a functional sensitivity of 0.3 ng/mL.

### Dx Imaging Protocol

Planar whole-body scans were performed 24 hours following the administration of 37 MBq I-131. Until 2010 a dual-headed Siemens Multispect-2 camera was used with 3 static images (head/neck, thorax/abdomen, pelvic/upper legs) of 10 min each, using a high-energy collimator and a 15% energy window centered at 364 KeV. From 2010 to 2015 imaging was performed on a Siemens Symbia S or T2 dual-headed gamma camera also with a high-energy collimator and the same centering of the energy window. Whole body planar images were recorded with a scan rate of 8 cm/min. The uptake at the original thyroid bed was measured by an external gamma-probe for 2 minutes (Isomed 2400, Nuklear Medizintecknik, Dresden, GmbH). After subtracting a 2-minute background measurement at the upper leg, the uptake was presented as percentage of the administered activity of 37 MBq I-131.

NSAIDs or corticosteroids were generally prescribed to patients with an uptake > 5% at the discretion of the treating physician to avoid radiation-induced inflammation and oedema in the neck. In patients with an uptake > 10%, multidisciplinary consultation was arranged immediately with the surgeon, endocrinologist, nuclear physician and radiologist to either perform additional surgery after diagnostic imaging (such as a neck ultrasound, a I-131 SPECT/CT scan, or a head and neck MRI scan) or to decrease the standard activity dose of I-131.

### Impact of Dx Imaging on Patient Management

To specify cases in which a Dx scan could be of value, we reported the actual changes in clinical management based on the outcomes of the Dx scan. A change in clinical management was defined as additional surgery or a change in the administered activity of I-131. The patient group who had changes in clinical management were compared with those without changes in management, by using logistic regressions. Minor changes like prescription of anti-inflammatory drugs were also described. Radiation-induced inflammation was defined as physical complaints in the neck region occurring after the administration of 37 MBq I-131 until 2 weeks after I-131 therapy. Information on prescription of drugs and physical complaints after I-131 therapy was extracted from the electronic patient files.

### Statistics

All data were presented as number (percentage) for categorical variables, median (inter quartile range, IQR) for skewed variables, and as mean (± standard deviation [SD]) for variables with a normal distribution. Odds ratios (OR) and 95% Confidence Intervals (CI) were calculated for each variable. The biomarkers TSH, free T4 and Tg were dichotomized. Cut-off values were obtained by maximizing the Youden-index with a receiver-operating characteristic (ROC) curve. Binary logistic regression models were used, in which variables with a univariable significance of p < 0.15 were added to a multivariable model. Each variable that did not contribute with a significance of p < 0.10 was incrementally removed from the multivariable model. The fit of the model was reported by Nagelkerke’s $R_{N}^{2}$. A p-value < 0.05 was considered statistically significant. Missing data were excluded from analysis. All statistical analyses were performed using the IBM Statistical Package for Social Science (SPSS version 23).

## Results

**Figure 1:**
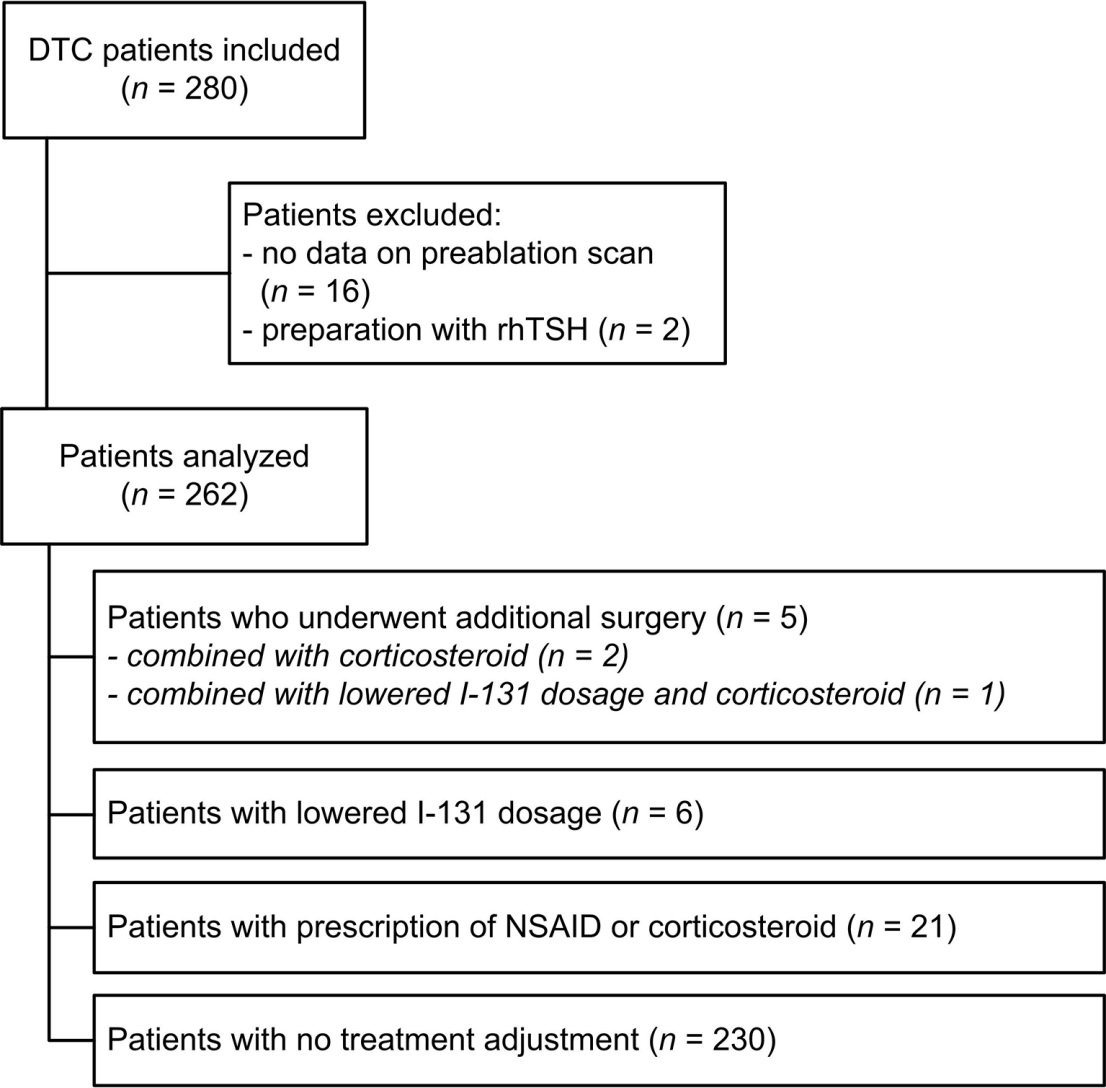
Flowchart of inclusion and clinical management. DTC, differentiated thyroid carcinoma; NSAID, non-steroidal anti-inflammatory drugs; rhTSH, recombinant human thyrotropin.

In total 280 patients underwent a total thyroidectomy in our region followed by treatment with I-131 at the UMC Groningen from 2005 to 2015. Two patients were excluded as they received I-131 therapy with recombinant TSH and in 16 patients the data of the Dx scan was missing, leaving 262 patients available for analysis (**Figure 1**). Baseline characteristics are shown in **Table 1**. Patients had an average age of 48.0 ± 17.7 years and 69% of patients were female. The median uptake in the thyroid bed was 2.4% (interquartile range 1.3-4.5%). The Dx scan showed additional findings in 99 patients (26.3%). The uptake was > 5% in 52 of 262 patients (19.8%) of which the uptake was > 10% in 15 patients (5.7%). A total of 17 out of 262 patients (6.5%) had no visible uptake in the original thyroid bed at the Dx scan and their uptake measurements varied between 0.1 and 2.3%. The Dx scan showed unexpected regional and distant metastases in 18 patients (7.3%). These 18 patients were already classified as high-risk according to the Dutch risk-stratification and the Dx scan did not alter the risk classification or administered I-131 activity. When using the simplified ATA-stratification, 6 of these 18 patients (33.3%) where classified as high risk and 12 (66.7%) as intermediate risk and the Dx scan also did not alter the ATA risk-stratification.

**Table 1:** Patient characteristics at DTC diagnosis.

	All patients (n = 262)
Mean age (years) ± SD	48.0 ± 17.7
Sex n (%)	
Male	81 (30.9)
Female	181 (69.1)
Histology n (%)	
Papillary	206 (78.6)
Follicular	35 (13.3)
Hürthle	21 (8.0)
Procedure of surgery n (%)	
One-step	141 (53.8)
Two-step	121 (46.2)
Location of surgery n (%)	
Tertiary	160 (72.5)
Non-tertiary	72 (27.5)
TNM Tumor stage^1^ n (%)	
Tx-T2	142 (54.2)
T3-T4	120 (45.8)
Nx-N0	145 (55.3)
N1	117 (44.7)
Mx-M0	240 (91.6)
M1	22 (8.4)
Dutch risk stratification n (%)	
Low risk	37 (14.1)
High risk	225 (85.9)
ATA risk stratification^2^ n (%)	
Low risk	87 (33.2)
Intermediate risk	143 (54.6)
High risk	32 (12.2)
AJCC Cancer Stage^1^ n (%)	
I	131 (50.0)
II	30 (11.5)
III	34 (13.0)
IV	67 (25.6)
Median TSH (mU/L) (IQR)	96.0 (66.25 - 131.0)

^1^AJCC TNM 7th edition.

^2^ATA risk stratification 2015.

DTC, differentiated thyroid carcinoma; SD, standard deviation; TSH, thyrotropin; IQR, interquartile range; ATA, American Thyroid Association.

### Change in Patient Management

In 11 patients (4.2%) the Dx scan changed clinical management, of which 4 patients required additional surgery (uptake ranging from 16 to 27%). The indication for additional surgery was confirmed with MRI of the neck region. A fifth patient with uptake of 42.9%, first underwent re-surgery. Thereafter, the uptake was still 23.9% resulting in the administration of a decreased activity of I-131. In these 5 patients, pathological examination after resurgery revealed benign thyroid tissue in 3 patients and in 2 patients DTC in a thyroid remnant with additional lymph node metastases was found. For 6 patients with an uptake between 5.6 and 24.0% the I-131 activity was reduced by 50% due to high uptake on the Dx scan. A further 6 patients (2.3%) had an uptake of >10% (range: 11.4-15.4%) but required no additional surgery or lower activity of I-131 at the discretion of the treating physician.

Patients with a change in clinical management, more often had a history of previous neck surgery compared to patients without a change in clinical management (**Table 2**: OR 5.9, 95% CI: 1.4-24.5). Of the 72 patients who underwent surgery in a non-tertiary hospital, 9 patients (12.5%) had a change in clinical management compared to 2 patients (0.1%) with first surgery in a tertiary hospital (OR 13.4, 95% CI: 2.8 – 63.8). TNM-stage, age, gender and ATA risk classification were not predictors of change of management after the Dx-scan. In a univariate model the biomarkers TSH <53.4 mU/L (OR 19.64, 95% CI: 4.94-78.13), thyroglobulin ≥50.0 ng/L (OR 7.4, 95% CI: 1.6-34.9) and free T4 ≥4.75 pmol/L (OR 156.8, 95% CI: 28.4-864.2) were all predictive of a change in clinical management. The regression model showed that free T4 >4.75 pmol/L alone is the strongest risk factor for a change in clinical management $\left(R_{N}^{2}\;0.549\right)$.

**Table 2:** Patient and treatment characteristics associated with a change in clinical management.

	Unchanged management(n = 251)	Change in management(n = 11)	Odds ratio	95%CI	
Age (10 years) mean ± SD	48.0 ± 18.0	47.0 ± 11.3	0.97	0.68 – 1.36	p=0.85
Sex n (%)					
Male	78 (31.1)	3 (27.3)	Ref.		
Female	173 (68.9)	8 (72.7)	1.20	0.31 – 4.65	p=0.79
Histology n (%)					
Papillary	197 (78.5)	9 (81.8)	Ref.		
Follicular	54 (21.5)	2 (18.2)	0.81	0.17 – 3.86	p=0.79
TNM Tumor stage n (%)					
T stage					
Tx-T2	133 (53.0)	9 (81.8)	Ref.		
T3-T4	118 (47.0)	2 (18.2)	0.25	0.05 – 1.18	p=0.08
N stage					
Nx-N0	137 (54.6)	8 (72.7)	Ref.		
N1	114 (45.4)	3 (27.3)	0.53	0.28 – 1.01	p=0.25
M stage					
Mx-M0	229 (91.2)	11 (100)	*		
M1	22 (8.8)	0			
ATA risk classification n (%)					
Low risk	80 (31.9)	7 (63.6)	Ref.		
Intermediate/high risk	171 (68.1)	4 (36.4)	0.27	0.08 – 0.94	p=0.04
History of neck surgery n (%)					
No	235 (94.0)	8 (72.7)	Ref.		
Yes	15 (6.0)	3 (27.3)	5.88	1.41 – 24.45	p=0.02
Procedure of surgery n (%)					
One-step	137 (54.6)	4 (36.4)	Ref.		
Two-step	114 (45.5)	7 (63.6)	2.10	0.60- 7.67	p=0.24
Location of first surgery n (%)					
Tertiary	188 (74.9)	2 (18.2)	Ref.		
Non-tertiary	63 (25.1)	9 (81.8)	13.43	2.83 – 63.81	p=0.001
TSH n (%)					
≥53.4 mU/L	30 (12.0)	8 (72.7)	Ref.		
<53.4 mU/L	221 (88.0)	3 (27.3)	19.64	4.94 – 78.13	p<0.001
Free T4 n (%)					
<4.75 pmol/L	244 (97.2)	2 (18.2)	Ref.		
≥4.75 pmol/L	7 (2.8)	9 (81.8)	156.8	28.4 – 864.2	p<0.001
Thyroglobulin n (%)					
<50.0 ng/L	156 (62.2)	2 (18.2)	Ref.		
≥50.0 ng/L	95 (37.8)	9 (81.8)	7.39	1.56 – 34.93	p=0.01

Odds ratios with 95% CI: and p-values were calculated with an univariable logistic regression. Follicular thyroid carcinoma included Hürtle cell carcinoma.

*Could not be assessed.

CI, confidence interval; DTC, differentiated thyroid carcinoma; ATA, American Thyroid Association; Ref., reference category.

Furthermore, there was no association between uptake measurement and results of re-stratification with a diagnostic I-131 whole body scan 6 months after I-131 therapy (OR 1.0, 95% CI: 0.9-1.1, p 0.891).

### Radiation-Induced Local Side Effects

The uptake was >5% in a total of 52 patients of which 21 patients (40.4%) were prescribed anti-inflammatory drugs, without a decision for further intervention. A total of thirteen patients (25.0%) with an uptake of >5% reported physical complaints during I-131 therapy. Five patients reported physical complaints during I-131 therapy despite using either NSAID’s or corticosteroids. A further 7 patients reported physical complaints without being treated with anti-inflammatory drugs. One patient reported physical complaints despite reduction of I-131 activity. The reported complaints consisted of pain (n = 5) and swelling (n = 3) at the original thyroid bed, and inflammation of the throat (n = 5).

## Discussion

The Dx scan is used prior to I-131 treatment to select patients for additional surgery, lower I-131 activity, or to consider prescribing anti-inflammatory drugs prior to I-131 treatment. This current analysis of 262 Dx scans shows that 26.3% of Dx scans revealed additional findings, like no uptake, high uptake (>5%), or unexpected regional or distant metastases. Other series have reported similar rates of unexpected findings ranging from 53% in a theoretical approach in 355 patients (6) to around 25% in other studies (7, 8). This would result in a ‘number needed to scan’ between approximately 2 to 4, which would certainly justify the regular use of a Dx scan. But while our current study confirmed a high incidence of additional findings, a change in clinical management was only seen in 4.2% of patients. Retrospectively, the Dx scan did not alter the patient’s risk class according to the Dutch risk classification and all of the patients with suggestion of regional lymph node or distant metastases on the Dx scan were ATA intermediate or high risk. In our study the Dx scan would therefore not have influenced the level of I-131 activity to be administered. Chen and colleagues also reported additional information in 25% of patients who underwent a Dx scan, but a change in clinical management only occurred in 5.7% of patients by changing the administration of I-131 in a two-step approach (8).

Besides limited clinical consequences, there are several other potential disadvantages linked to the Dx scan, including low sensitivity compared to the post-Tx scan and the risk of stunning. Stunning is a radiobiological phenomenon defined as a temporary suppression of the iodine trapping function of the thyrocytes and thyroid cancer cells as a result of the radiation given off by the first, low dose of I-131. Stunning can be avoided by using I-123 or by lowering the activity of I-131 to 37 MBq as in our current protocol (13).

Both the limited effect on clinical management and the potential disadvantages imply a selected use of the Dx scan. To assist in selecting patients for a Dx scan, we identified several pre-scan risk factors for a change in management. High plasma levels of Tg and free T4, and low levels of TSH were associated with a higher likelihood of a change in management. Moreover, patients who had undergone first surgery in a non-tertiary center or had a history of previous neck surgery had a higher likelihood of a change in management, suggesting a relation with surgical approach and/or quality. Our study included patients from various low- and high-volume surgical centers, allowing to compare the risk for a change in management, based on surgical centers. The uptake on the Dx scan has previously also been reported to be lower after one-step thyroidectomy (versus two-step) and after surgery by more experienced surgeons (14, 15). Thus, the value of the Dx scan is largely determined by the risk of a thyroid remnant and thus quality of surgery. This last interpretation cannot be confirmed with our data, because we did not register parameters on quality of surgery. The outcome of thyroid surgery, in terms of complications and length of hospital stay, is also better with a higher volume operated by a thyroid surgeon (9, 16–18). So, while in general the Dx scan has limited influence on clinical management, it could be considered based on history of the patient, previous surgical outcomes in a center, and potentially on levels of Tg and free T4.

A major limitation of this study is the non-protocolled retrospective design. Six patients with an uptake of >10% did not have a change in clinical management at discretion of the treating physician. This could result in an underestimation of the effect of the Dx scan, but these small number of patients do not significantly influence the results of the study. Referral bias could also result in a higher rate of management change for patients referred from low-volume centers, but because only our center treats DTC patients in the region with I-131 this effect is also very limited.

Using the Dx scan to deescalate treatment by lowering or even withholding I-131 could again increase the value of the Dx scan. This cannot be studied with the design of our study but has been reported earlier. In a study by Avram and colleagues. the Dx scan was involved in individualizing I-131 activities (19). The Dx scan was used to stratify patients on risk of recurrence. Subsequently, dosimetry calculations were performed based on whole body uptake counts and blood activity counts. This approach resulted in a complete response after a single RAI therapy in 88% of patients without distant metastases, opening a new potential approach to the Dx scan.

## Conclusion

The Dx scan before RAI therapy in patients with differentiated thyroid cancer has a low yield regarding change of clinical management. Selective use of the Dx scan in patients with a medical history of previous neck surgery or surgically treated in a in low-volume hospital may have advantages. Whether to perform a Dx-scan should be considered based on patient characteristics and prior center-based surgical outcomes.

## Data Availability Statement

The original contributions presented in the study are included in the article/supplementary material. Further inquiries can be directed to the corresponding author.

## Ethics Statement

Ethical review and approval was not required for the study on human participants in accordance with the local legislation and institutional requirements. Written informed consent for participation was not required for this study in accordance with the national legislation and the institutional requirements.

## Author Contributions

All authors contributed to the article and approved the submitted version. TB and WZ analyzed the data and wrote the manuscript with input from all authors.

## Conflict of Interest

The authors declare that the research was conducted in the absence of any commercial or financial relationships that could be construed as a potential conflict of interest.
